# Community-Acquired Pseudomonas aeruginosa Meningitis in a Pediatric Patient

**DOI:** 10.7759/cureus.42376

**Published:** 2023-07-24

**Authors:** Alexander Cotran-Lenrow, Lidia S Tefera, Matthew Douglas-Vail, Arnold Ayebare, Leroy N Kpokpah, Bill P Davis

**Affiliations:** 1 Department of Microbiology, Partners in Health, Harper, LBR; 2 Department of Pediatrics, Partners in Health, Harper, LBR; 3 Department of Emergency Medicine, University of British Columbia, Vancouver, CAN; 4 Department of Pediatrics, James Jenkins (JJ) Dossen Hospital, Ministry of Health, Harper, LBR

**Keywords:** pseudomonas aeruginosa, meningitis, gram-negative meningitis, pseudomonas aeruginosa meningitis, pediatric bacterial meningitis

## Abstract

This case report presents a rare and significant case of community-acquired *Pseudomonas aeruginosa* meningitis in a healthy 13-month-old male patient in rural Liberia. *Pseudomonas aeruginosa* meningitis, particularly in the absence of predisposing factors, is a rare occurrence with a high mortality rate. The challenges in diagnosing this condition, especially in resource-limited settings, are highlighted. The patient initially presented with fever, seizures, and altered consciousness, and lumbar puncture revealed turbid cerebrospinal fluid (CSF) with elevated white blood cell count. Subsequent CSF culture confirmed *Pseudomonas aeruginosa* infection. Prompt initiation of appropriate antibiotic therapy, including a push dose of meropenem, resulted in clinical improvement. However, the patient exhibited post-meningitis sequelae, including hearing and visual impairments. Comprehensive follow-up care and rehabilitation services are crucial for managing these long-term complications. By sharing this case, we aim to increase awareness and facilitate early recognition of* Pseudomonas aeruginosa* meningitis, leading to improved patient care and outcomes in similar clinical scenarios.

## Introduction

Bacterial meningitis is a severe infection characterized by inflammation of the meninges, the protective membranes surrounding the brain and spinal cord [1]. Meningitis is a medical emergency that requires prompt recognition and treatment to prevent significant morbidity and mortality. The most common bacterial culprits vary by age group, with *Group B Streptococcus* being most common in infants less than two months, while *Streptococcus pneumoniae* is the most common in all other age groups except for ages 11-17, where *Neisseria meningitides* is the most common [1]. Infections with gram-negative bacilli such as *Escherichia coli, Klebsiella, Enterobacter*, and *Pseudomonas aeruginosa *are less common, and account for only 7% of cases [2]. Among this already small group, *Escherichia coli* and *Pseudomonas* species are the most prevalent pathogens [2]. One recent case series lasting 18 years found that 19 out of 21 patients with *P. aeruginosa* found in cerebrospinal fluid (CSF) had undergone neurosurgical procedures, had extra ventricular devices, or had previously been colonized with *Pseudomonas* [3].

Patients with Gram-negative bacilli meningitis (GNBM), particularly with *Pseudomonas aeruginosa*, face a severe clinical picture - of the etiological agents, GNBM cases have the highest fatality rate at 54% [4]. The incidence and mortality rates of bacterial meningitis vary across different regions and populations, with a higher prevalence observed in low-income countries and areas with limited access to healthcare facilities. Therefore, the occurrence of *Pseudomonas aeruginosa *meningitis in the absence of any predisposing factors is an exceptionally rare and intriguing finding.

This case report aims to provide a comprehensive overview of the clinical course, diagnostic challenges, treatment strategies, and ultimate prognosis of community-acquired *Pseudomonas aeruginosa* meningitis in a healthy 13-month-old male patient in rural Liberia. By shedding light on this atypical case, we hope to enhance awareness and facilitate early recognition of *Pseudomonas meningitis*, ultimately contributing to improved patient care and outcomes in similar clinical scenarios.

## Case presentation

An otherwise healthy 13-month-old boy presented to the JJ Dossen emergency room in Harper, Liberia with his mother, complaining of two days of high-grade fever and seizure-like activity. The mother claimed the seizures occurred more than five times per day and that the child had no history of prior recurrent or serious infections, including ear and sinus infections. Notably, the patient had received Pneumococcal and Haemophilus influenzae vaccines, however, he had not received the Meningococcal vaccine as it is not part of the childhood vaccination series in Liberia. At presentation, his pulse rate was 157 beats/min, respiratory rate 44 breaths/min, febrile to 38°C, and oxygen saturation 93%. On physical exam, he was ill-appearing, had pale conjunctiva and palmar pallor, responded only to noxious stimuli, and looked and moaned when painful stimuli were elicited. His anthropometric parameters were in normal range as a result the possibility of malnutrition was ruled out. A lumbar puncture was performed and cerebrospinal fluid (CSF) was sent for analysis and culture. Blood cultures were also drawn. The patient was subsequently started on weight-based phenytoin for seizure control, ceftriaxone for presumed bacterial meningitis, and IV artesunate for cerebral malaria. Neuroimaging was considered but was not available in the rural Liberian setup. Later he was transferred to the Pediatrics ward after stabilization.

On admission, initial investigations revealed an elevated white blood cell count of 17.2 x 10^9^ cells/L and Hemoglobin of 7.7 g/dl, which is low for his age. Liver function tests and coagulation screens were normal. The cerebrospinal fluid appeared slightly yellow and turbid, showed a markedly increased WBC count (400 cells/mm^3^), normal protein levels (30 mg/dL) and the glucose level was 250 mg/dL, but was not compared to serum glucose. The gram stain of the CSF did not show any bacterial pathologies. Rapid malaria and HIV diagnostics were negative and given the lack of prior recurrent or serious infections, underlying immunodeficiency was ruled out, although we were unable to perform further testing to evaluate for less common underlying immunosuppression.

**Figure 1:**
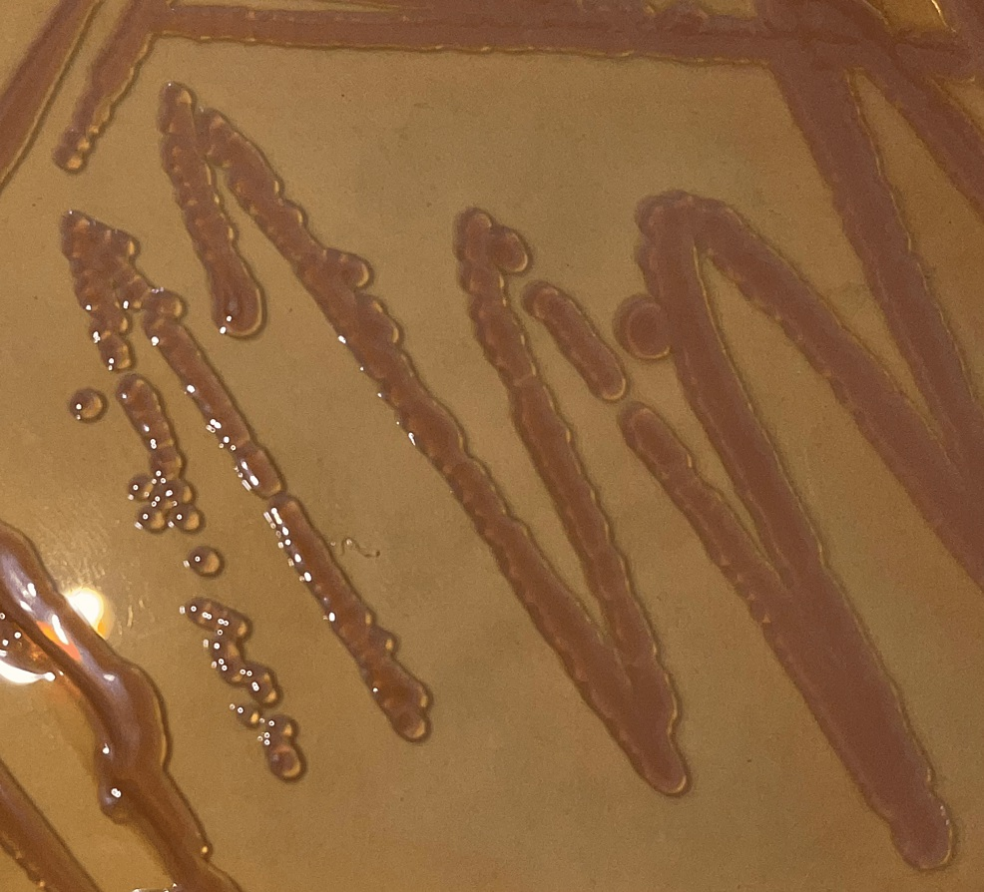
Pseudomonas aeruginosa colony morphology on MacConkey agar isolated from CSF CSF: Cerebrospinal Fluid

The following day, the CSF culture was positive for *Pseudomonas aeruginosa*, determined phenotypically (Figure 1) and by Remel RapID NF (Thermo Fisher Scientific, Waltham, MA, USA) biochemical assay. Blood cultures were negative after 48 hours. The preliminary results were released to the clinician, and a drug susceptibility by disc diffusion was performed. Empiric antibiotic therapy and IV treatment for cerebral malaria was stopped. As a precaution, the patient was started on meropenem monotherapy prior to susceptibility results due to the high incidence of intrinsic resistance associated with *Pseudomonas* species. Despite the known side effects of meropenem, the local availability of antibiotics and high rates of resistance in the region made it the most appropriate choice of antibiotic. Given the severity of the clinical picture at the time of the culture result and the lack of clinical improvement after ceftriaxone administration, the first dose of meropenem was given as a push dose. 400mg of meropenem was administered over three minutes. This reduced the time-to-first-dose which was deemed necessary for this patient given his ongoing fever, seizures, and leukocytosis. Once the antimicrobial susceptibility test (Table 1) was confirmed, a 21-day regimen of meropenem was initiated (Table 2).

**Table 1:** Antimicrobial susceptibility results of the Pseudomonas aeruginosa isolated from CSF S: Susceptible, I: Intermediate, R: Resistant, CSF: Cerebrospinal Fluid

Antibiotic	Interpretation
Cefepime	S
Ciprofloxacin	S
Trimethoprim/Sulfamethoxazole	R
Gentamycin	R
Piperacillin-Tazobactam	S
Meropenem	S

**Table 2:** Clinical time course of the patient with Pseudomonas aeruginosa meningitis CRO: Ceftriaxone, ART: Artesunate, MEM: Meropenem

D1	D2	D3	D4	D5	D6	D7+
Cultures Pending		Pseudomonas aeruginosa +				
CRO+ART		MEM				
Fever						
Seizure						

During his stay in the pediatric ward, his vital signs and neurologic conditions were monitored. The patient gained consciousness and had marked improvement in his clinical condition. After 48 hours, the child's fever and seizure activity subsided. The patient completed a three-week course of antibiotics and was discharged with ferrous sulfate and an appointment for a follow-up visit at one month. On discharge, he exhibited signs of post-meningitis sequelae. He did not respond to calls, high-intensity or low-pitched sounds, and does not blink to visual stimuli. Unfortunately, otolaryngologists and ophthalmologists are not available in the region, and he could not have eye and hearing assessments.

## Discussion

Meningitis caused by *Pseudomonas aeruginosa* is a rare occurrence, particularly in the absence of predisposing factors such as previous neurosurgical procedures, specialized CSF drains, or prior infection with the bacteria [3]. The case presented here is of significant interest as it describes an atypical case of *Pseudomonas aeruginosa *meningitis in an otherwise healthy 13-month-old child in a rural Liberian setting. Accurate diagnosis of *Pseudomonas aeruginosa* meningitis can be challenging, especially in resource-limited settings where advanced diagnostic tools may not be readily available. In this case, the initial gram stain of the cerebrospinal fluid (CSF) did not reveal any bacterial pathogens, highlighting the limitations of relying solely on this method for diagnosis. In addition, gram stains are negative in 60-80% of untreated cases of bacterial meningitis. The presence of blood in the CSF can further complicate gram-stain results [5]. Comprehensive culture and sensitivity testing are crucial for accurately identifying the causative agent [6]. The positive CSF culture for *Pseudomonas aeruginosa* in this case emphasizes the importance of following up with culture results to guide appropriate antibiotic therapy and underscores the need for such capacity, even in remote settings.

Prompt initiation of appropriate antibiotic therapy is also crucial in the management of* Pseudomonas aeruginosa* meningitis. Once the pathogen was identified, the patient was first given a push dose of meropenem to reduce the time to first dose. We feel that this was essential in the patient’s positive prognosis. The choice and duration of antibiotic treatment may vary based on local resistance patterns but in this case, a 21-day course of meropenem was indicated, resulting in a marked improvement in the patient's clinical condition.

The prognosis of *Pseudomonas aeruginosa* meningitis is generally poor and is influenced by several factors, including the timely initiation of appropriate antibiotic therapy, the presence of predisposing factors, and the development of complications [4]. While the patient, in this case, showed improvement in his clinical condition, he exhibited signs of post-meningitis sequelae, specifically hearing and visual impairments. These sequelae may result from direct damage to the cranial nerves or other neurological structures during the course of the infection. Comprehensive follow-up care and rehabilitation services are essential for individuals who survive bacterial meningitis, as they may require long-term support and management of these sequelae [7]. While most of these services are not available in our setting, the patient was scheduled to be visited in the community by our community-health team and will be monitored for recurrent infection by the clinical team.

Our case report adds to the limited body of literature on this topic. The challenges in diagnosing and treating this condition described in previous studies align with our own observations, emphasizing the need for heightened clinical awareness and timely interventions [4,8]. By sharing this case with the medical community, healthcare providers can become more vigilant in considering *Pseudomonas aeruginosa* as a potential pathogen even in the absence of predisposing factors. Increased awareness can lead to earlier diagnosis, appropriate management, and improved outcomes for patients in similar clinical scenarios.

## Conclusions

This case report highlights the rarity and clinical significance of *Pseudomonas aeruginosa* meningitis in the absence of predisposing factors. The diagnostic challenges associated with this condition underscore the need for comprehensive culture and sensitivity testing. Prompt initiation of appropriate antibiotic therapy, such as meropenem, is crucial for improving patient outcomes. Long-term sequelae, including hearing and visual impairments, may be observed, emphasizing the importance of comprehensive follow-up care. By reporting this case, we aim to increase awareness and facilitate early recognition of *Pseudomonas aeruginosa* meningitis, ultimately contributing to improved patient care and outcomes in similar clinical scenarios.
